# Supplementary material for: Anti-Inflammatory Properties of Novel 1,2-Benzothiazine Derivatives and Their Interaction with Phospholipid Model Membranes
Source: Membranes (Basel). 2024 Dec 18;14(12):274. doi: 10.3390/membranes14120274 (PMC11678778; doi:10.3390/membranes14120274)
Supplement: Supplementary file 1 [file membranes-14-00274-s001.zip › membranes-3341914-supplementary.pdf]

Supplementary materials

Anti-inflammatory properties of novel 1,2-benzothiazine derivatives and their interaction with phospholipid model membranes

Berenika M. Szczęśniak-Sięga [1,*], Jadwiga Maniewska [1] , Benita Wiatrak [2], Tomasz Janek [3], Paulina Nowotarska [4] and Żaneta Czyżnikowska [5,*]

[1] Department of Medicinal Chemistry, Faculty of Pharmacy, Wroclaw Medical University, Borowska 211, 50-556 Wrocław, Poland; berenika.szczesniak-siega@umw.edu.pl (B.S.-Sz.); jadwiga.maniewska@umw.edu.pl (J.M.)
[2] Department of Pharmacology, Faculty of Medicine, Wroclaw Medical University, J. Mikulicza-Radeckiego 2, 50-345 Wrocław, Poland; benita.wiatrak@umw.edu.pl (B.W.)
[3] Department of Biotechnology and Food Microbiology, Faculty of Biotechnology and Food Science, Wroclaw University of Environmental and Life Sciences, Chełmońskiego 37, 51-630 Wroclaw, Poland; tomasz.janek@upwr.edu.pl (T.J.)
[4] Department of Biostructure and Animal Physiology, Wroclaw University of Environmental and Life Sciences, Norwida 25/27, 50-375 Wroclaw, Poland
[5] Department of Basic Chemical Sciences, Faculty of Pharmacy, Wroclaw Medical University, Borowska 211a, 50-556 Wroclaw, Poland; zaneta.czyznikowska@umw.edu.pl (Ż.C.)

* Correspondence: zaneta.czyznikowska@umw.edu.pl (Ż.C.) and berenika.szczesniak-siega@umw.edu.pl (B.S.-Sz.)

Content:

1. **^{1}H NMR** and **^{13}C NMR** spectra of new compounds (pages S3-S10).

2. Computational studies (pages S11-S17)

^{1}H NMR and ^{13}C NMR spectra of new compounds

compound	structure	page
**BS23**	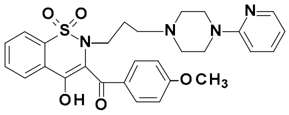	S3
**BS24**	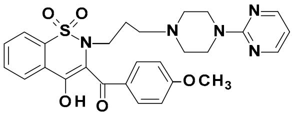	S4
**BS25**	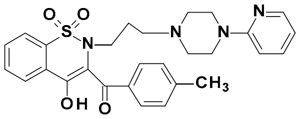	S5
**BS26**	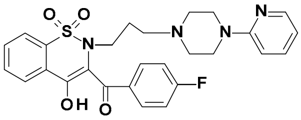	S6
**BS27**	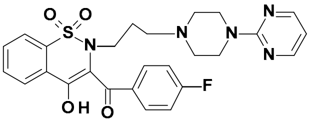	S7
**BS28**	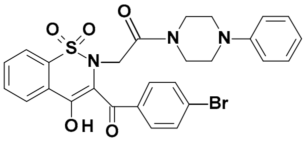	S8
**BS29**	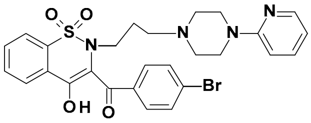	S9
**BS30**	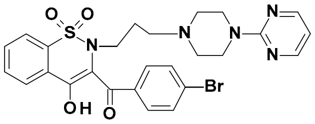	S10

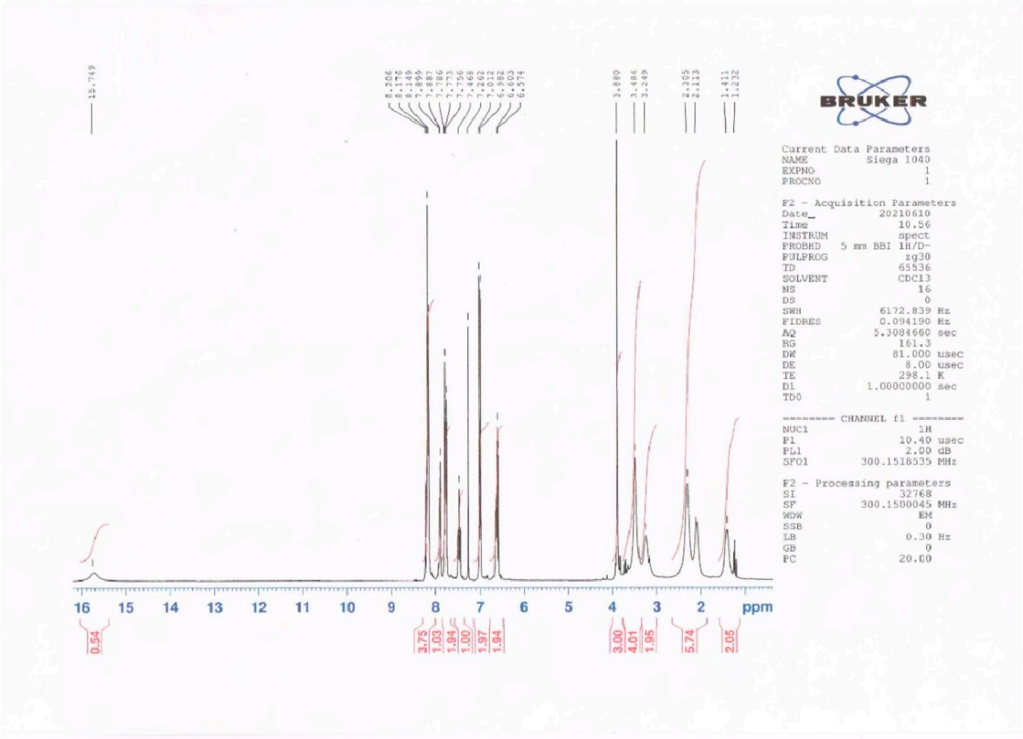

Figure S1. ^{1}H NMR spectrum of **BS23** in $CDCl_3$.

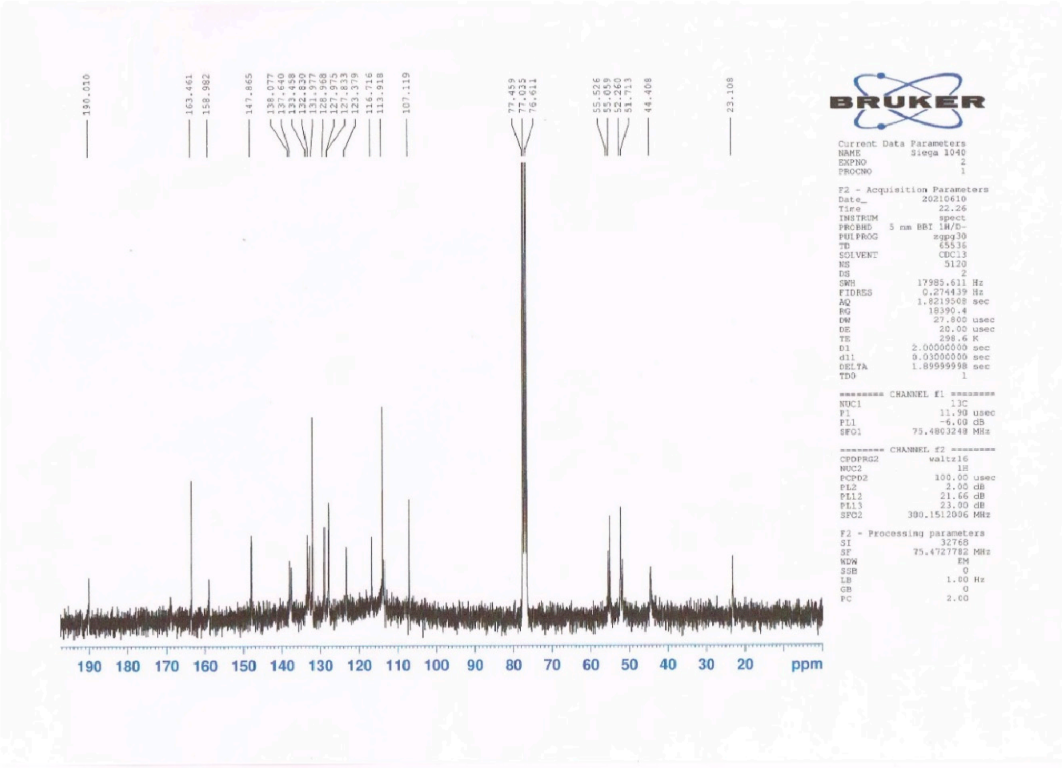

Figure S2. ^{13}C NMR spectrum of **BS23** in $CDCl_3$.

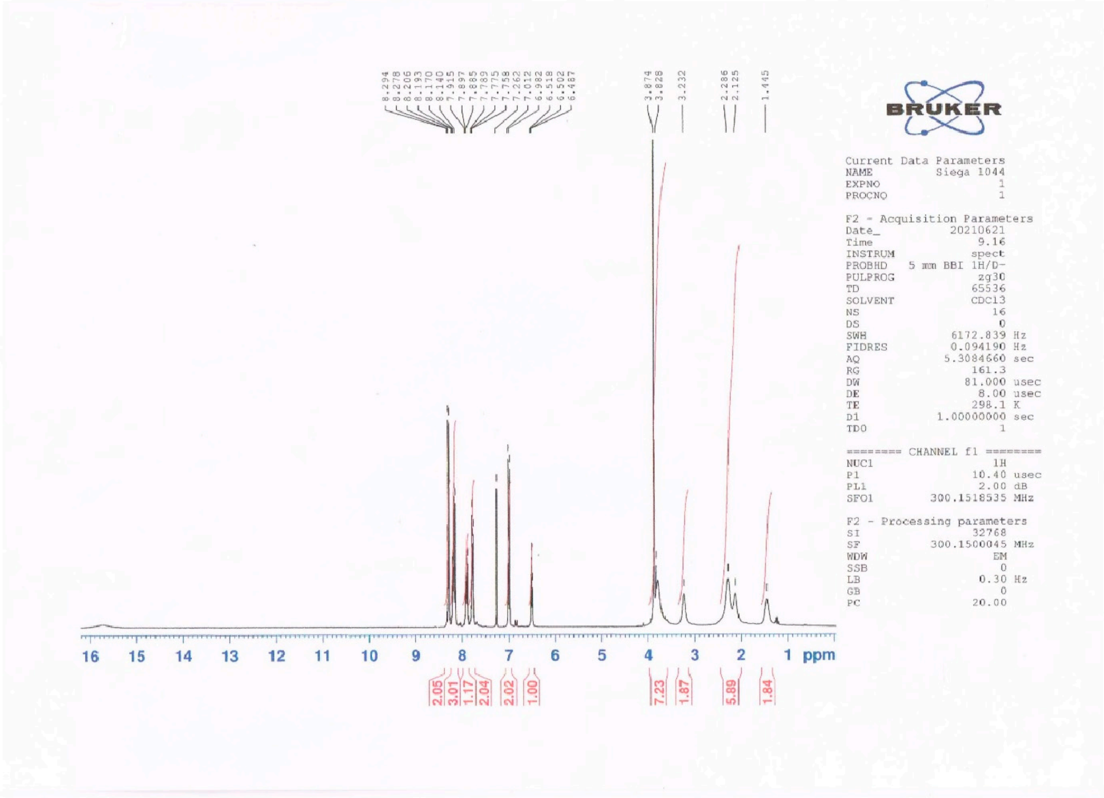

Figure S3. ^{1}H NMR spectrum of **BS24** in $CDCl_3$.

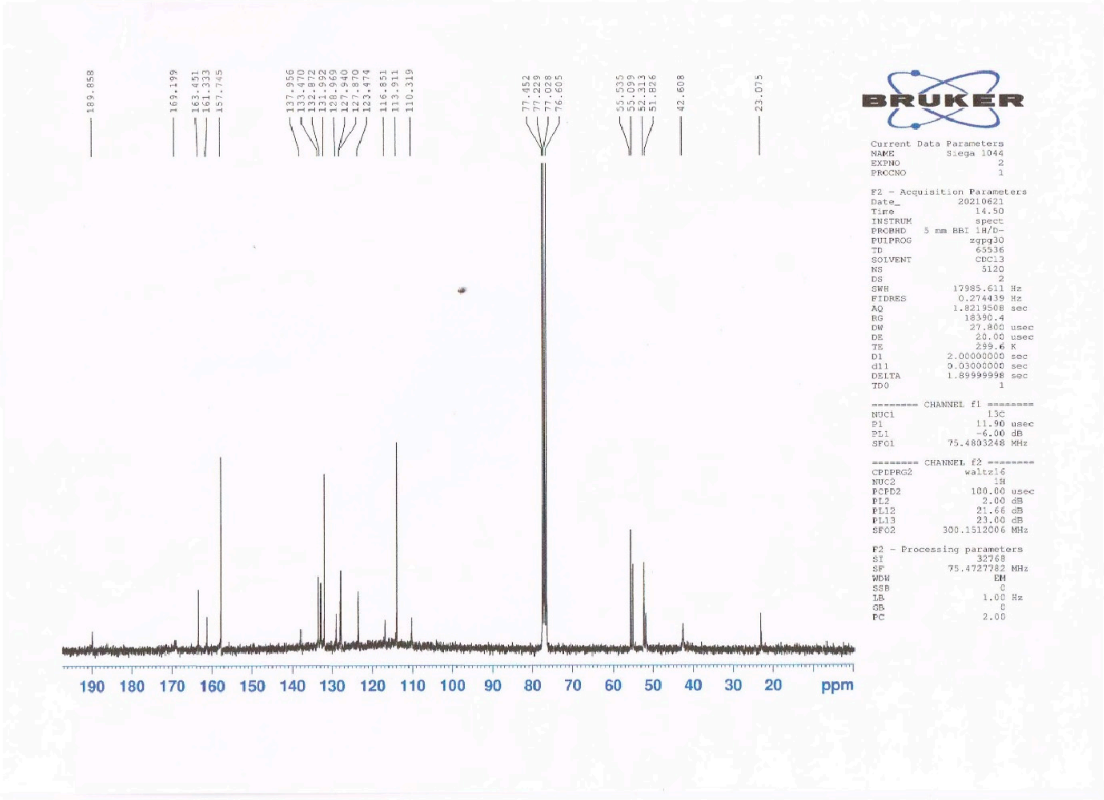

Figure S4. ^{13}C NMR spectrum of **BS24** in $CDCl_3$.

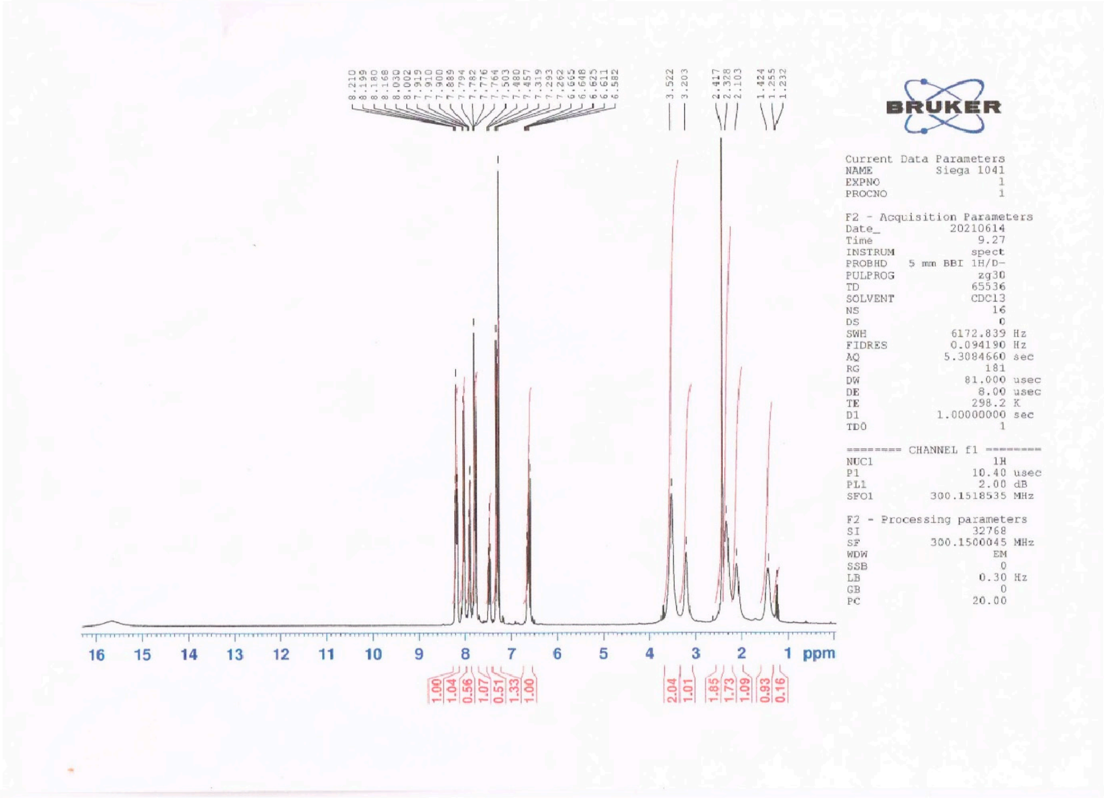

Figure S5. ^{1}H NMR spectrum of **BS25** in $CDCl_3$.

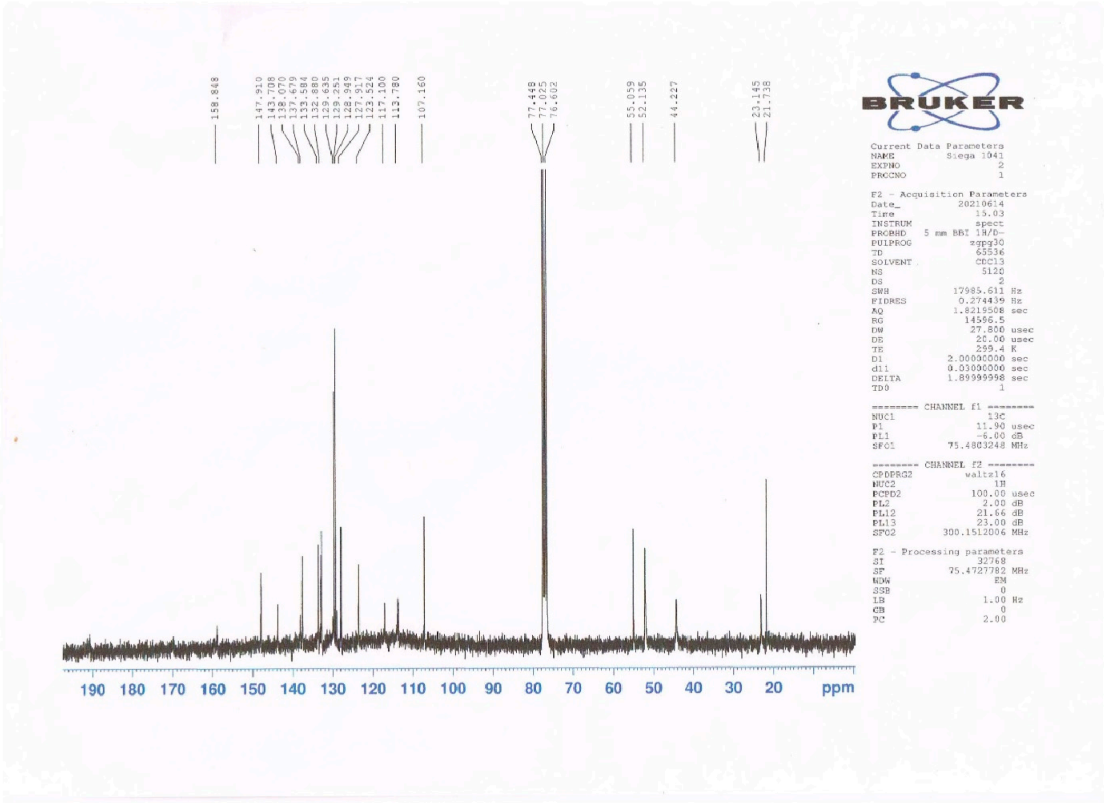

Figure S6. ^{13}C NMR spectrum of **BS25** in $CDCl_3$.

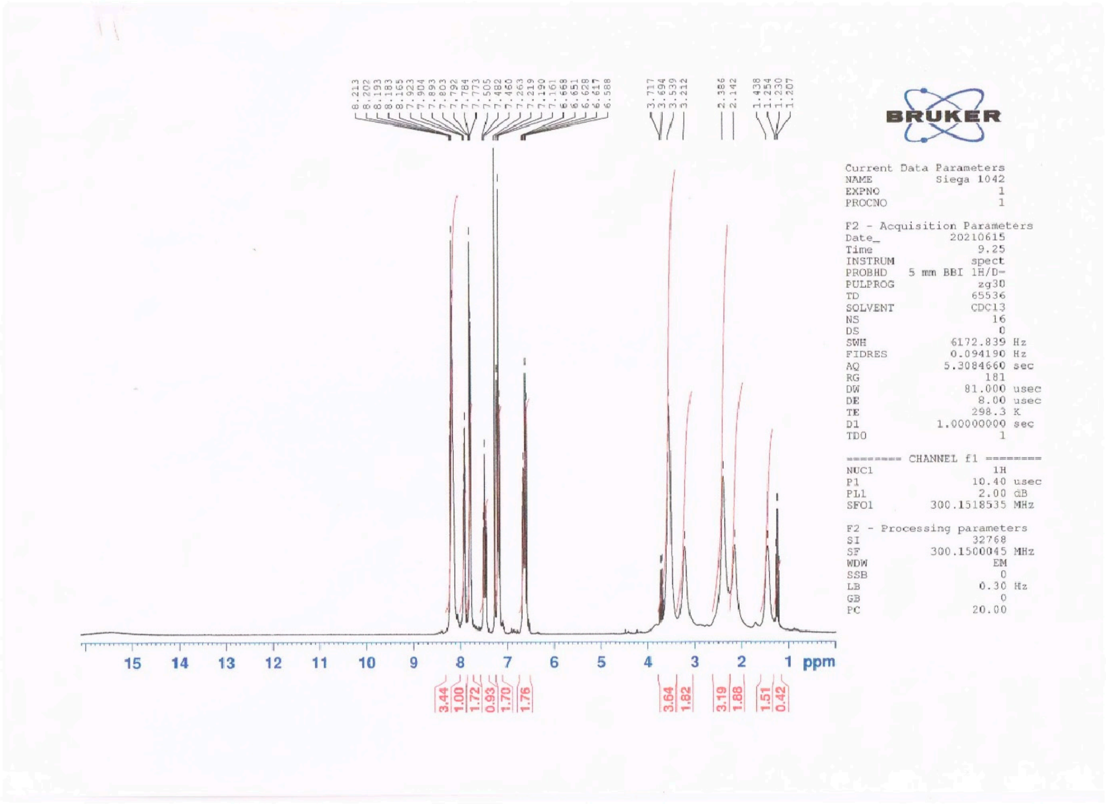

Figure S7. ^{1}H NMR spectrum of **BS26** in $CDCl_3$.

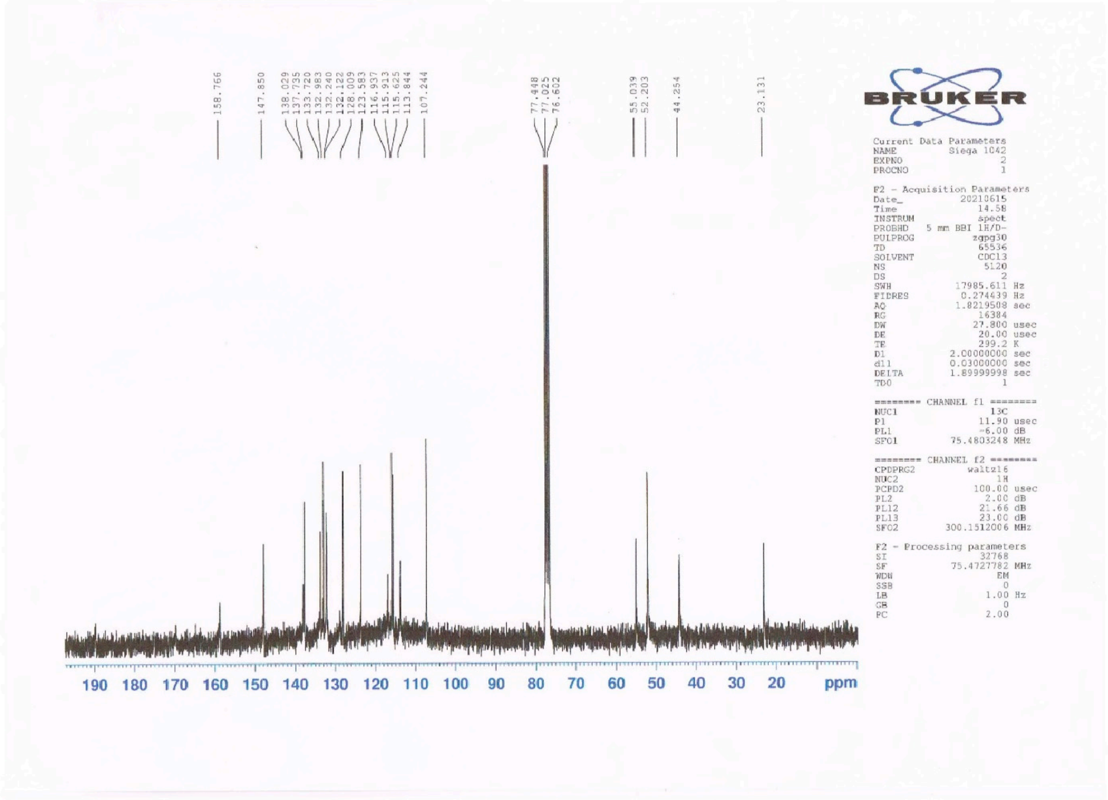

Figure S8. ^{13}C NMR spectrum of **BS26** in $CDCl_3$.

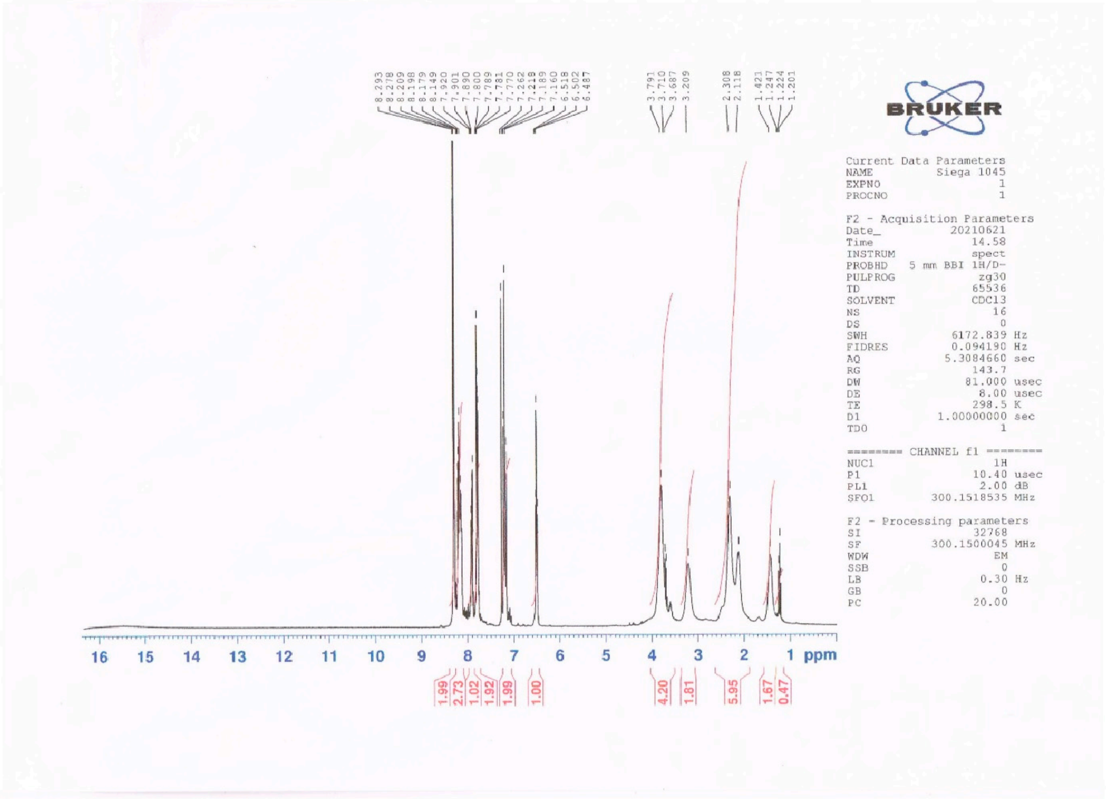

Figure S9. ^{1}H NMR spectrum of **BS27** in $CDCl_3$.

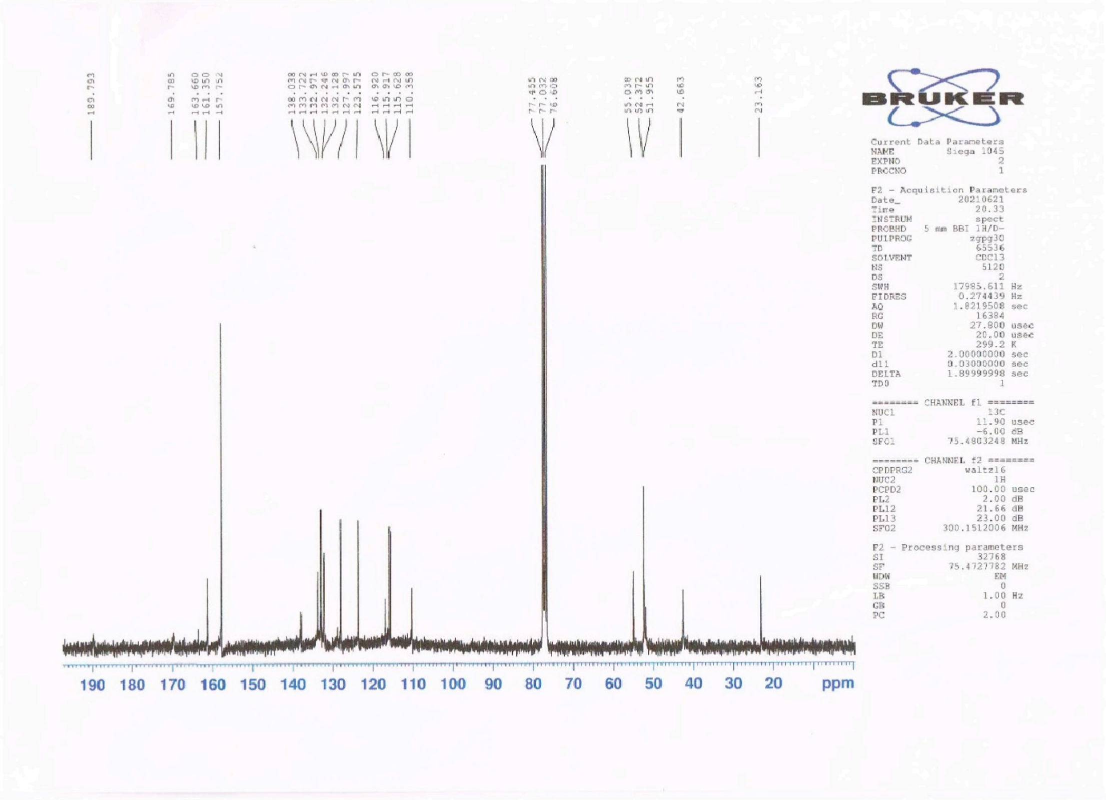

Figure S10. ^{13}C NMR spectrum of **BS27** in $CDCl_3$.

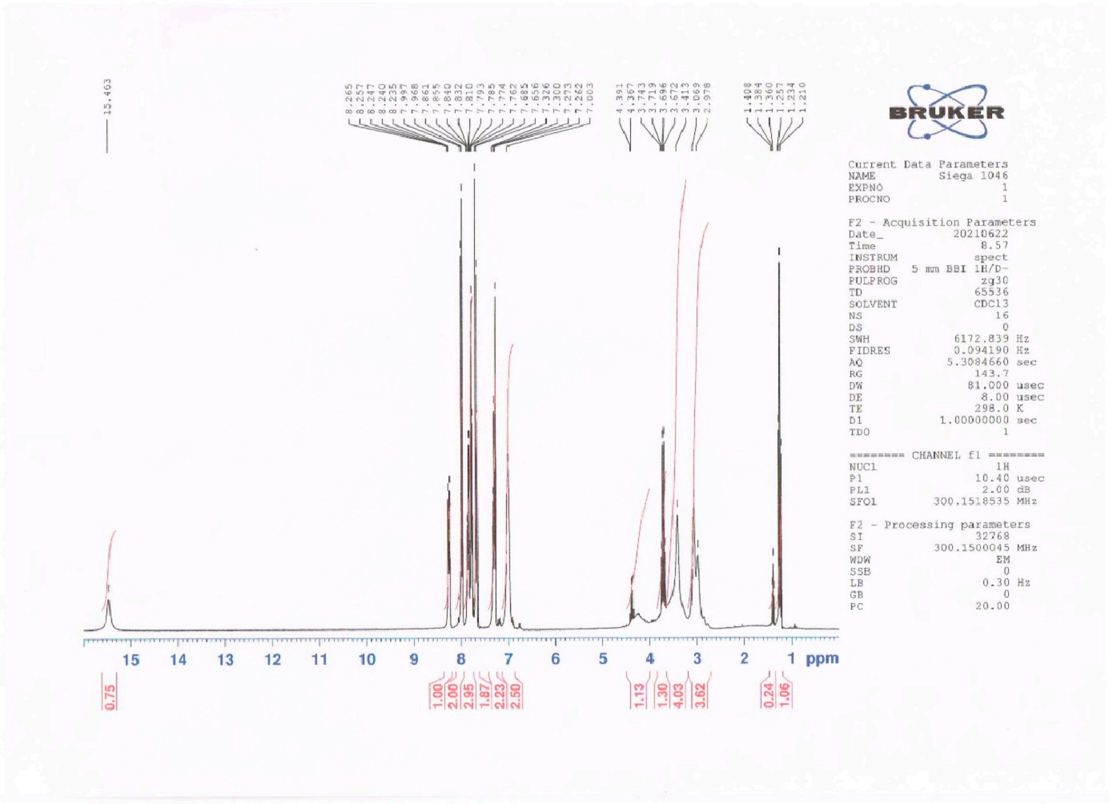

Figure S11. ^{1}H NMR spectrum of **BS28** in $CDCl_3$.

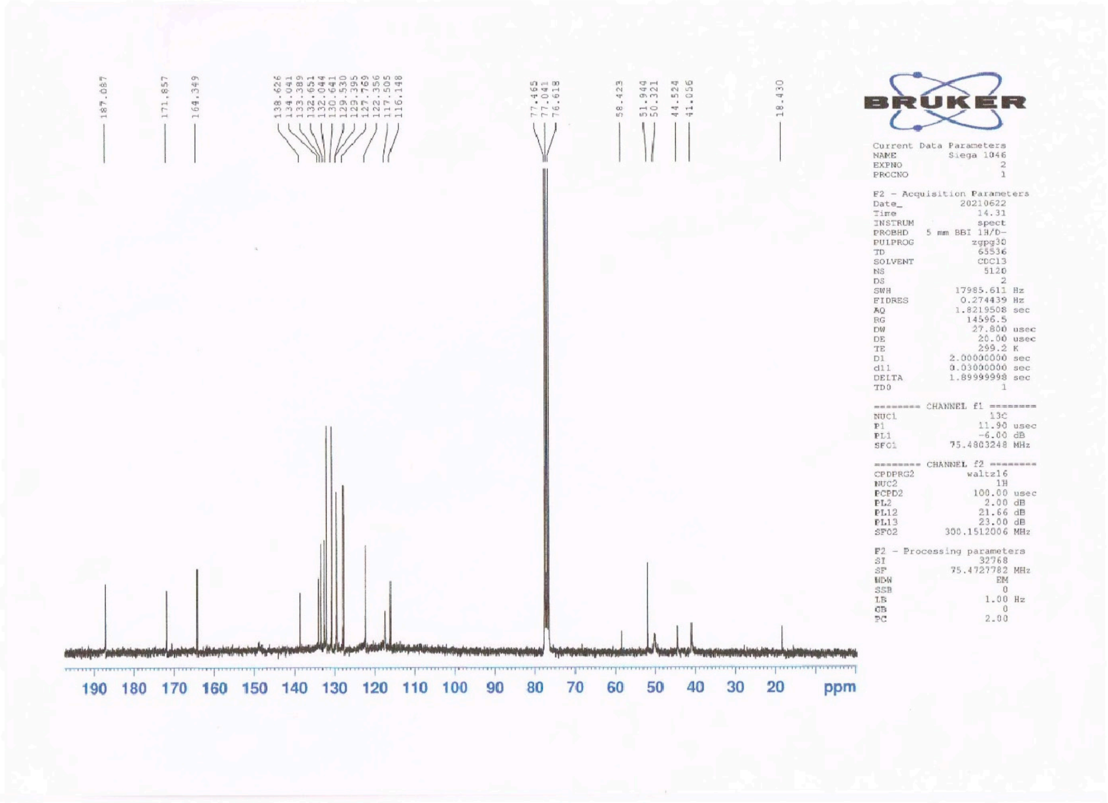

Figure S12. ^{13}C NMR spectrum of **BS28** in $CDCl_3$.

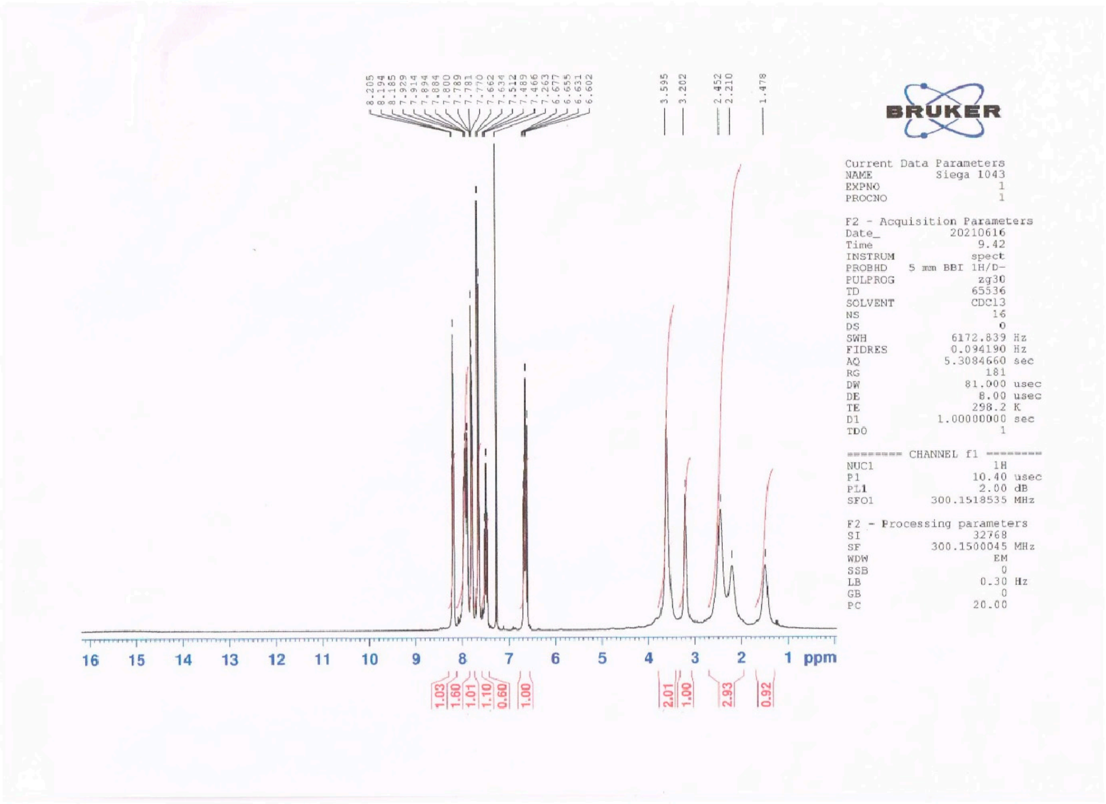

Figure S13. ^{1}H NMR spectrum of **BS29** in $CDCl_3$.

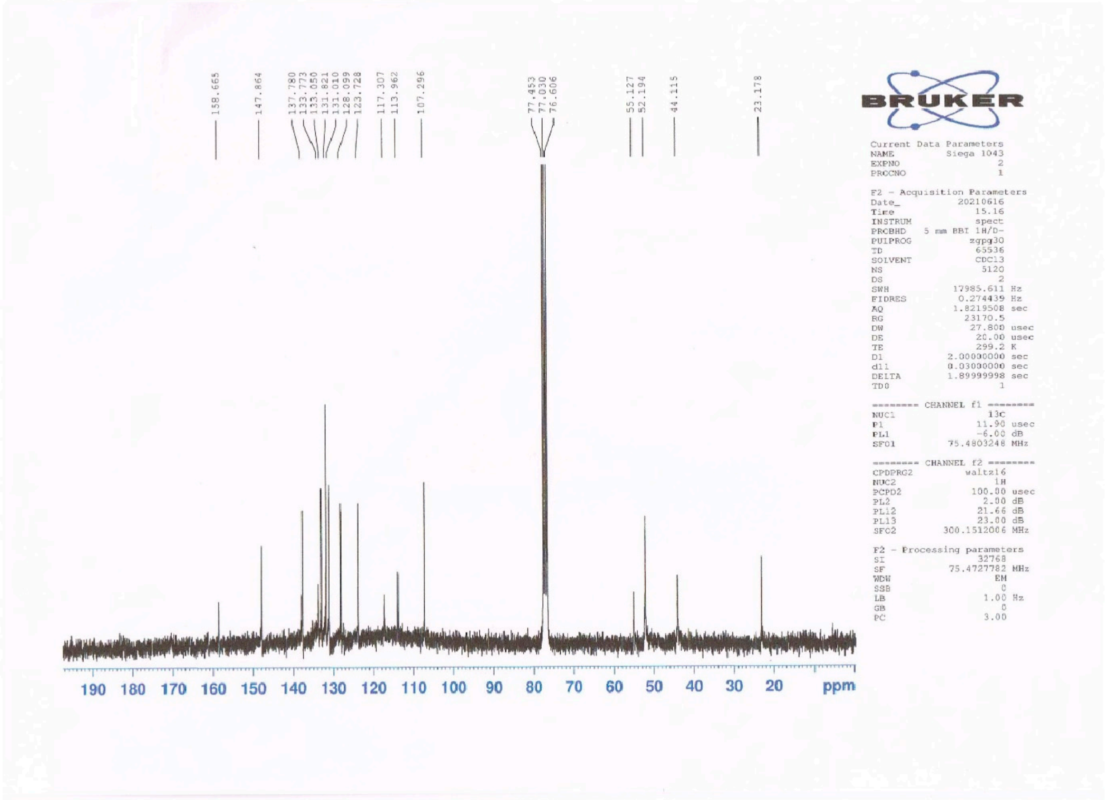

Figure S14. ^{13}C NMR spectrum of **BS29** in $CDCl_3$.

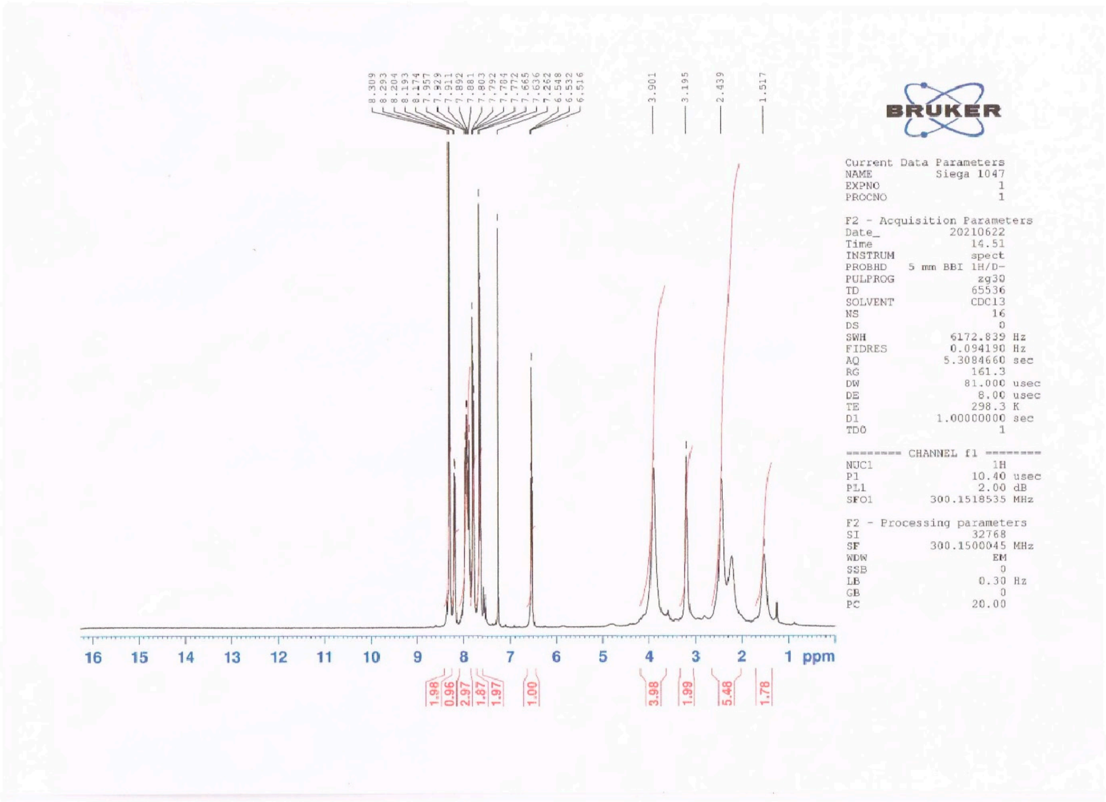

Figure S15. ^{1}H NMR spectrum of **BS30** in $CDCl_3$.

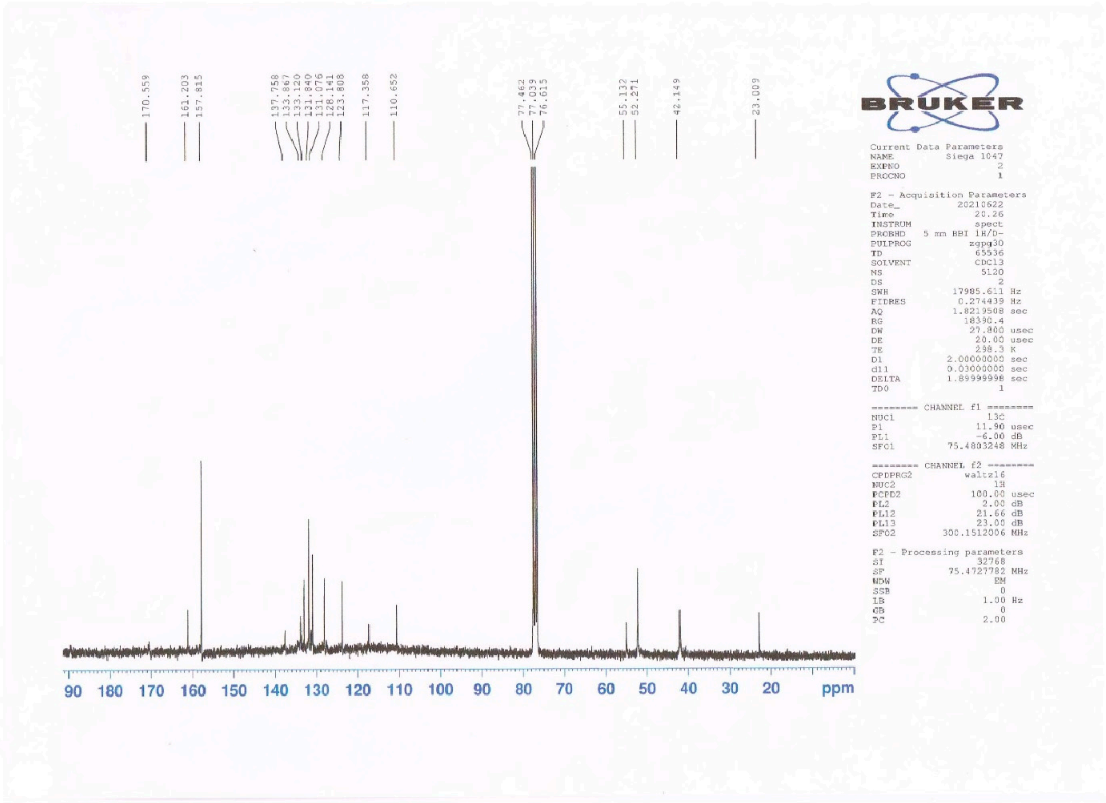

Figure S16. ^{13}C NMR spectrum of **BS30** in $CDCl_3$.

	HOMO	LUMO
**BS23**	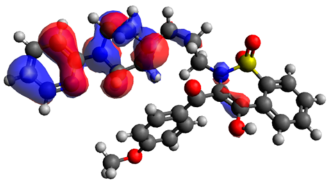	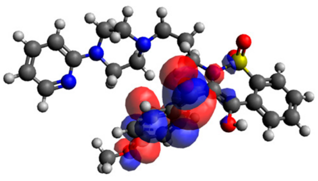
**BS24**	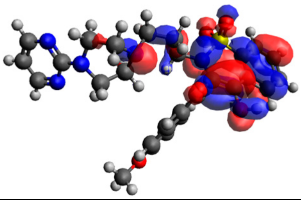	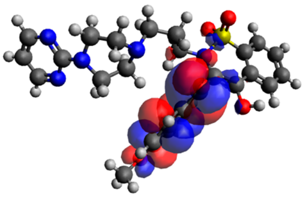
**BS25**	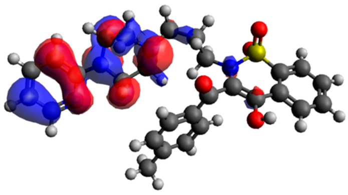	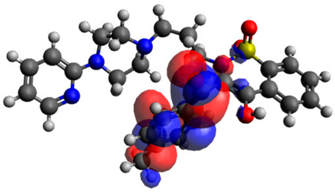
**BS26**	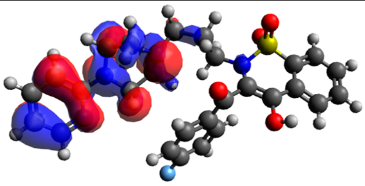	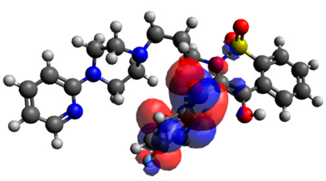
**BS27**	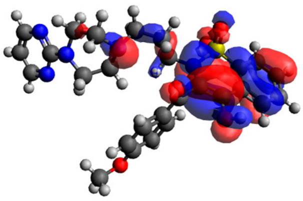	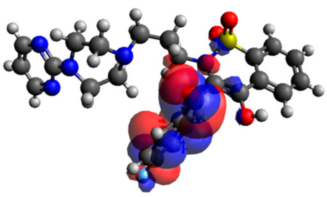
**BS28**	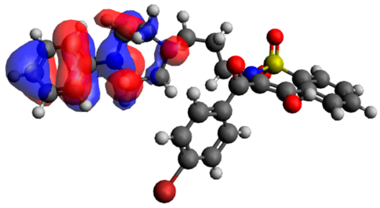	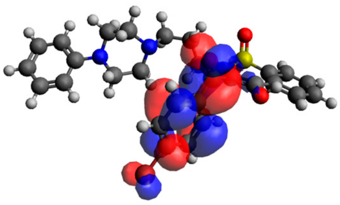
**BS29**	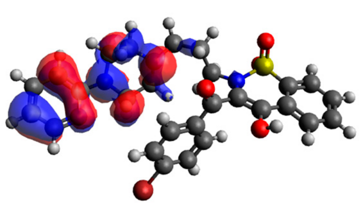	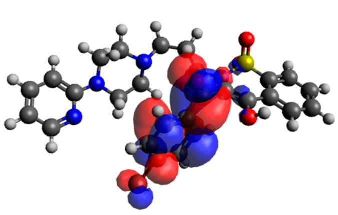
**BS30**	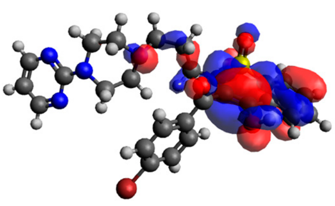	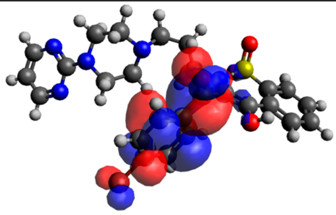

Figure S17. The distribution map of HOMO/LUMO densities. The negative electron density is presented in blue color while the red color indicates the positive electron density.

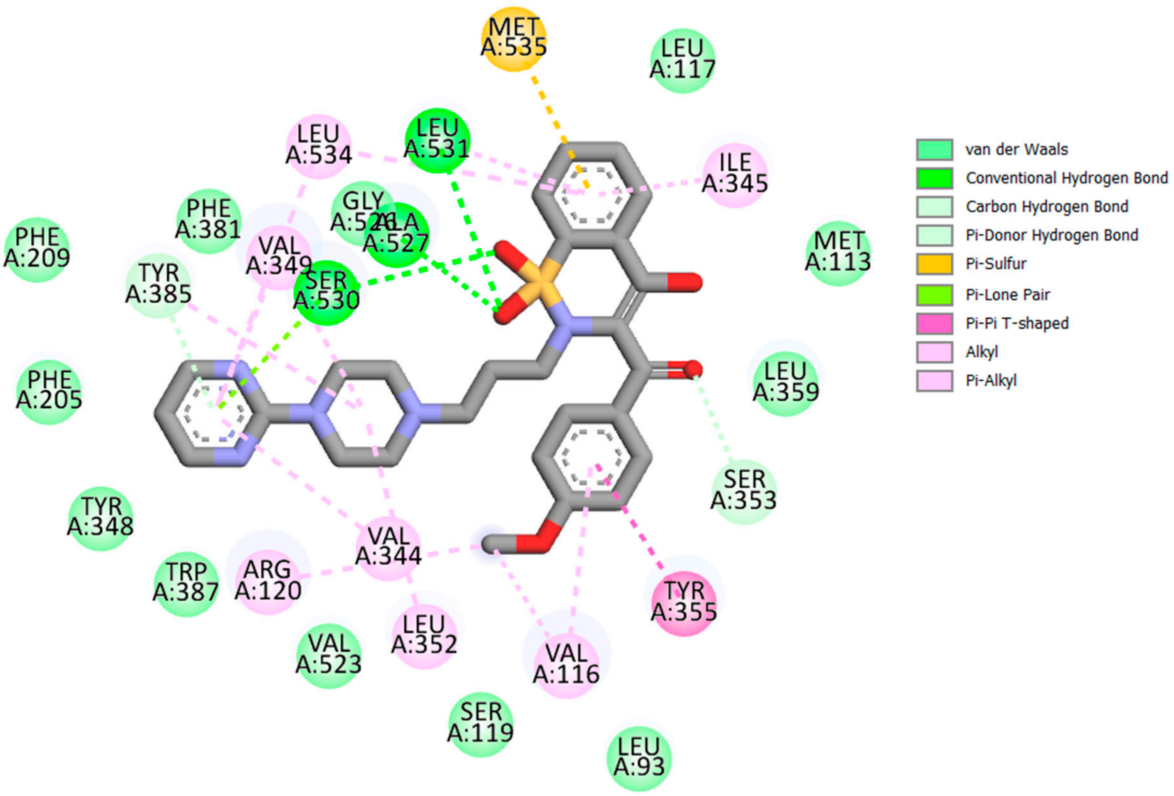

Figure S18. The binding mode of designed compound **BS24 – COX-2** in the active center of COX-2

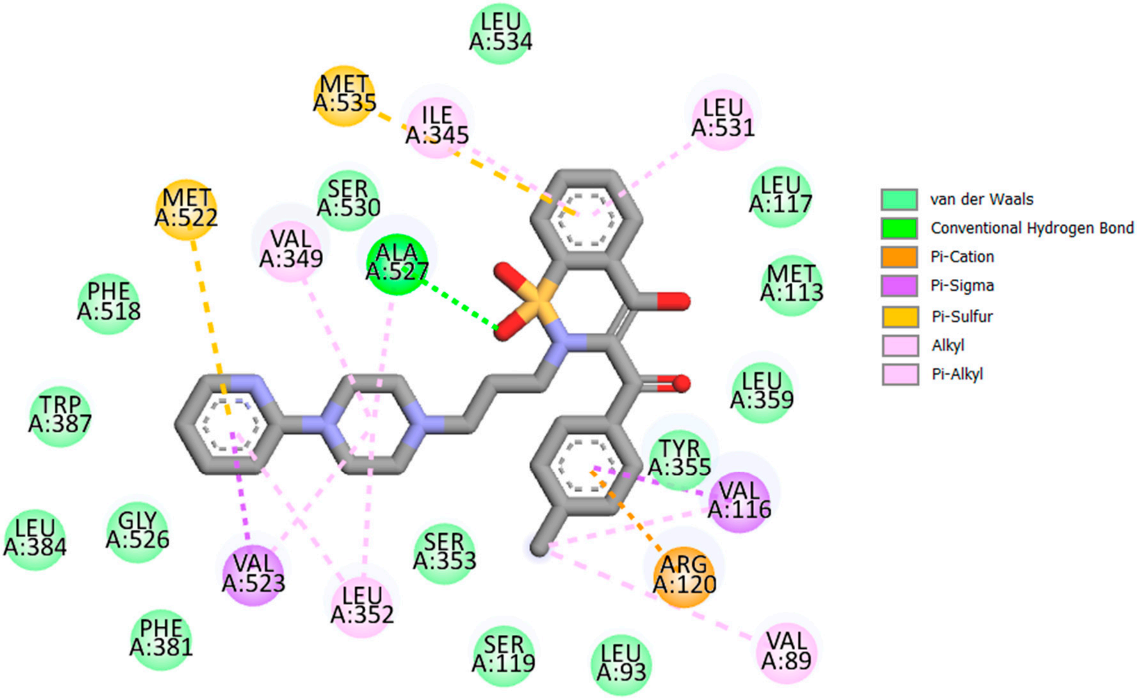

Figure S19. The binding mode of designed compound **BS25 – COX-2** in the active center of COX-2

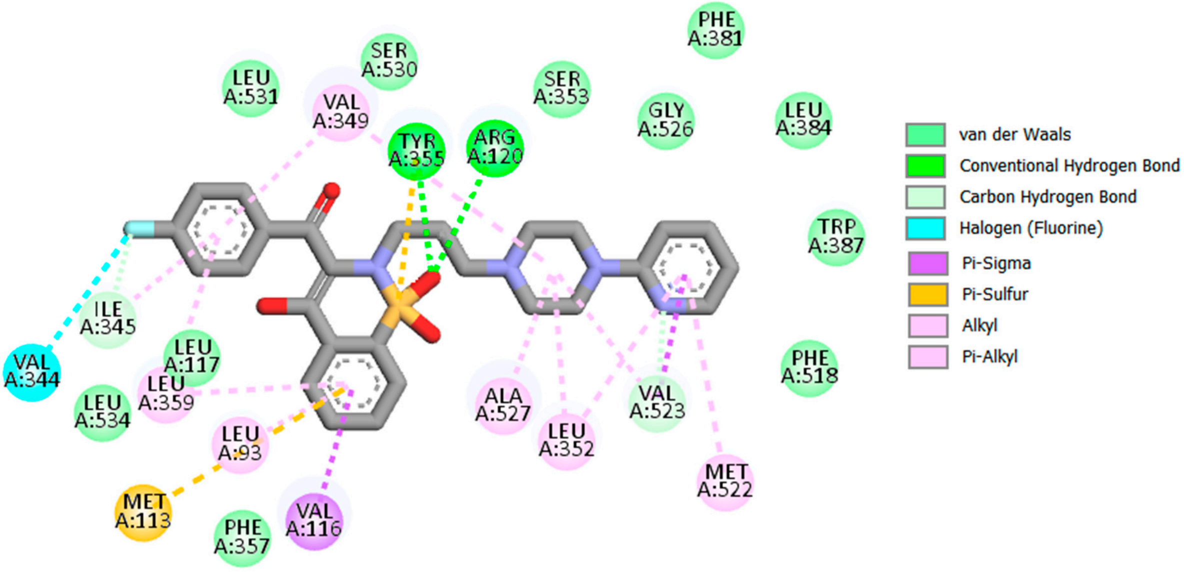

Figure S20. The binding mode of designed compound **BS26 – COX-2** in the active center of COX-2

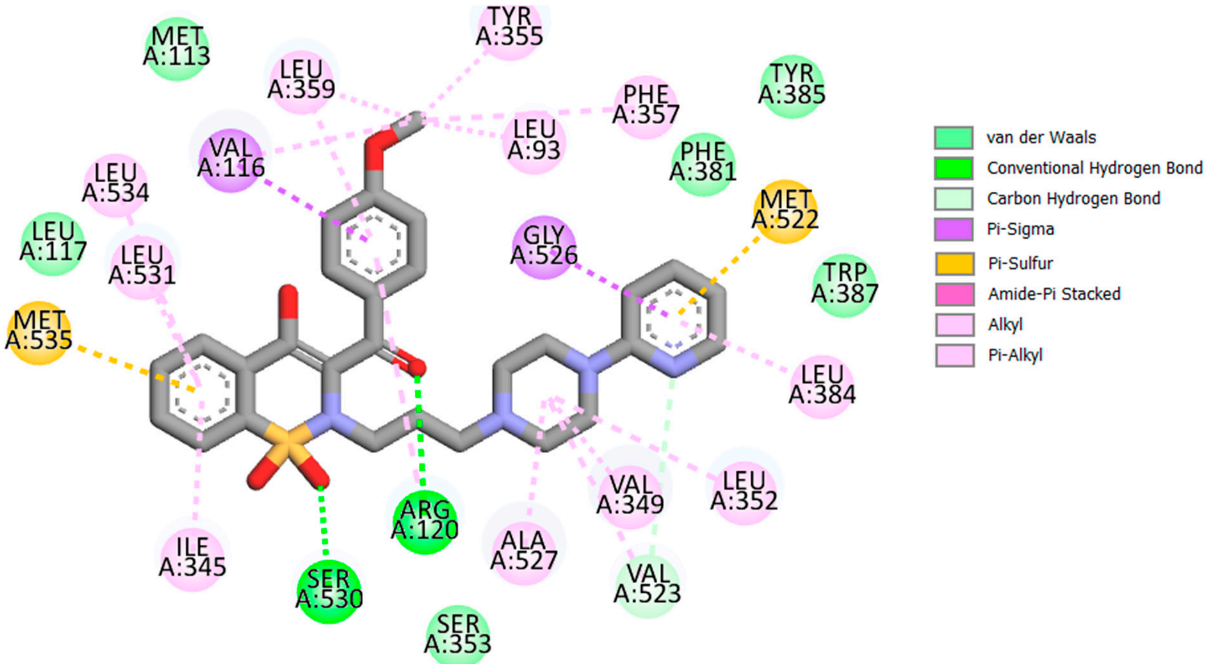

Figure S21. The binding mode of designed compound **BS27 – COX-2** in the active center of COX-2

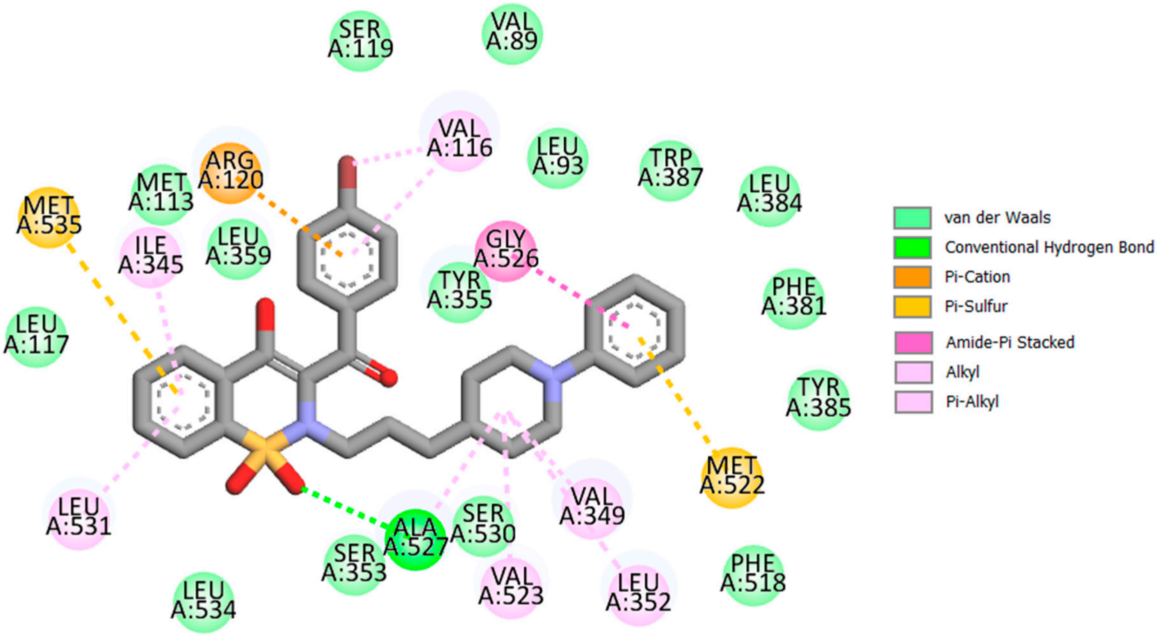

Figure S22. The binding mode of designed compound **BS28 – COX-2** in the active center of COX-2

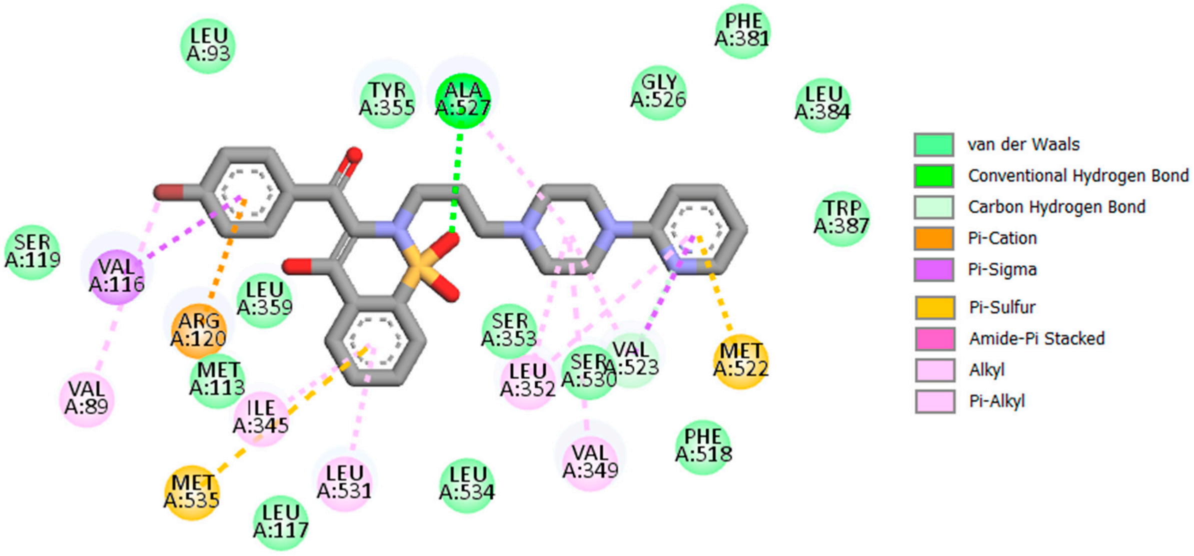

Figure S23. The binding mode of designed compound **BS29 – COX-2** in the active center of COX-2

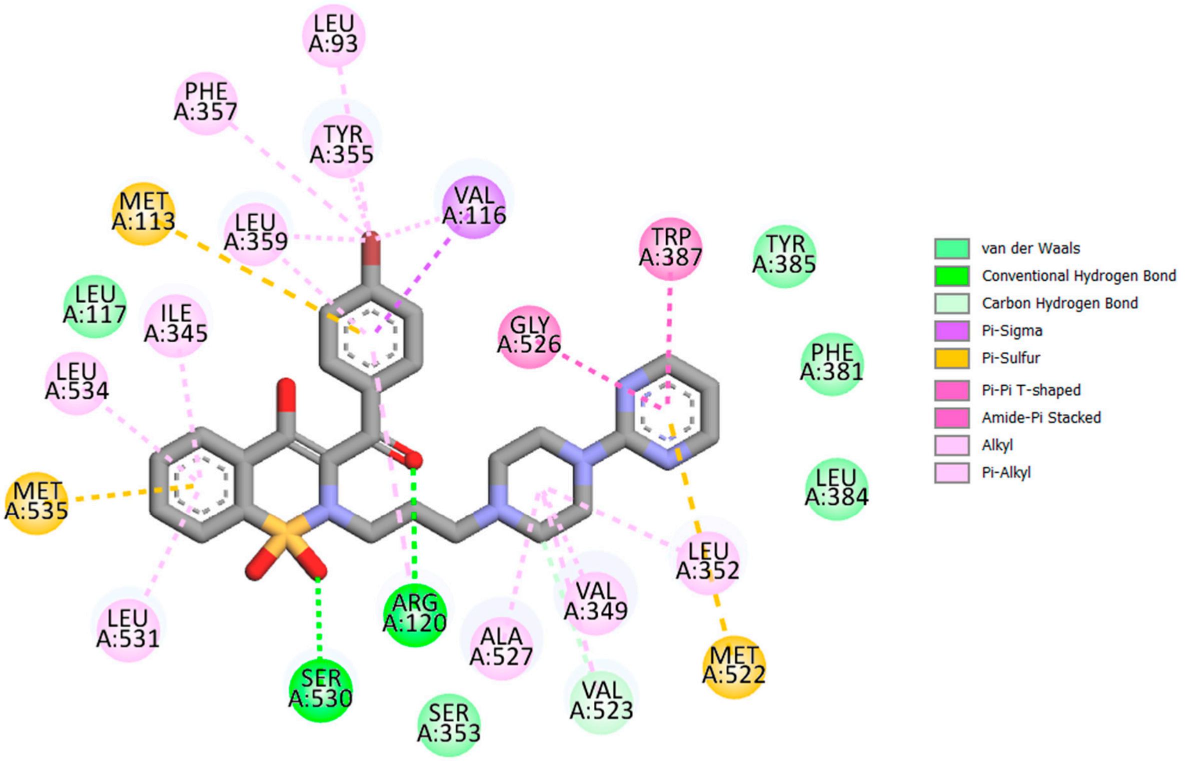

Figure S24. The binding mode of designed compound **BS30 – COX-2** in the active center of COX-2

Table S1. Intermolecular interactions in the active center of COX-1.

	$\Delta G_{binding}$ [kcal/mol]	van der Waals	hydrogen bonds	π-type interactions
**BS23**	-13.9	Ser353, Phe361, Phe518, Gly526, Leu534	Arg120, Ala527, Ser530, Tyr355	Leu93, Ile89, Met113, Val16, Leu117, Ile345, Leu357, Ile523, Leu535
**BS24**	-13.4	Ser353, Phe361, Phe518, Leu534, Gly526	Arg120, Tyr355, Ala527, Leu531	Ile89, Leu93, Val116, Ile345, Tyr355, Leu357, Leu359, Ile523, Leu531
**BS25**	-14.9	Met113, Leu117, Arg120, Phe321, Leu359, Trp387, Leu534, Leu535,	Ser353, Ala527	Val116, Ile345, Tyr355, Leu384, Tyr385, Met522, Gly526, Leu531
**BS26**	-13.9	Ser353, Phe361, Phe518, Leu534, Gly526	Ser530	Ile345, Leu357, Ile523, Leu531, Leu535
**BS27**	-14.1	Leu93, Arg120, Leu357, Tyr385, Phe381, Trp387, Ser530	Ser353, Ala527	Vla116, Leu117, Ile345, Tyr355, Leu384
**BS28**	-12.9	Met113, Arg120, Ser353, Leu357, Trp387, Leu535, Leu543	Ala527	Val116, Leu117, Ile345, Tyr355, Tyr385, Met522, Leu531,
**BS29**	-14.5	Arg120, Ser353, Leu357, Leu359, Phe381, Tyr385, Trp387, Phe518, Leu534, Leu535	Ala527	Leu93, Met113, Val116, Leu117, Ile345, Leu348, Tyr355, Leu384, Met522, Gly526, Leu531,
**BS30**	-13.8	Ile89, Met113, Arg120 Tyr348, Leu359, Phe381, Tyr385, Gly526, leu534, Leu535	Ser353, Ala527, Ser530, Leu531	Leu117, Val116, Ile345, Tyr355, Trp387, Leu531,
